# Heart Rate Variability as a Potential Indicator of Cancer Pain in a Mouse Model of Peritoneal Metastasis

**DOI:** 10.3390/s22062152

**Published:** 2022-03-10

**Authors:** Yurim Kim, Hong Yeol Yoon, Il Keun Kwon, Inchan Youn, Sungmin Han

**Affiliations:** 1 Department of Dentistry, Graduate School, Kyung Hee University, 26 Kyungheedae-ro, Dongdaemun-gu, Seoul 02447, Korea; yurim1501@naver.com (Y.K.); kwoni@khu.ac.kr (I.K.K.); 2 Biomedical Research Division, Korea Institute of Science and Technology, 5, Hwarang-ro 14-gil, Seongbuk-gu, Seoul 02791, Korea; seerou@kist.re.kr; 3 Department of Dental Materials, School of Dentistry, Kyung Hee University, 26 Kyungheedae-ro, Dongdaemun-gu, Seoul 02447, Korea; 4 Division of Bio-Medical Science & Technology, KIST School, Korea University of Science and Technology (UST), Seoul 02792, Korea

**Keywords:** pain, autonomic nervous system, heart rate variability, electrocardiogram, peritoneal metastasis

## Abstract

Heart rate variability (HRV) is closely related to changes in the autonomic nervous system (ANS) associated with stress and pain. In this study, we investigated whether HRV could be used to assess cancer pain in mice with peritoneal metastases. At 12 days after cancer induction, positive indicators of pain such as physiological characteristics, appearance, posture, and activity were observed, and time- and frequency-domain HRV parameters such as mean R-R interval, square root of the mean squared differences of successive R-R intervals, and percentage of successive R-R interval differences greater than 5 ms, low frequency (LF), high frequency (HF), and ratio of LF and HF power, were found to be significantly decreased. These parameters returned to normal after analgesic administration. Our results indicate that overall ANS activity was decreased by cancer pain and that HRV could be a useful tool for assessing pain.

## 1. Introduction

Pain is an abnormal sensation of the body alerts that is usually caused by an injury, localized tissue damage, or illness [1]. There are different types of pain: acute pain, which lasts for a short period of time and goes away when the cause is treated, and chronic pain, which persists for more than six months and can be the underlying cause of diseases such as rheumatoid arthritis, osteoarthritis, fibromyalgia, multiple sclerosis, and cancer [2,3,4]. In many cases, chronic pain is very complex and can progress to a psychological level after the physical problem has healed. Therefore, the treatment and management of chronic pain have a great impact on quality of life.

Pain assessment is important for providing analgesia, and a numerical rating scale (NRS) or visual analog scale (VAS) have been considered good clinical practice to quantify pain in patients [5]. Although these methods are becoming common tools for measuring pain intensity, there are still limitations, such as individual variations, validity of correlation between specific diseases and pain intensity, and inability to apply in noncommunicating patients. Therefore, several methods have been investigated for pain assessment, including heart rate variability (HRV) analysis [6]. HRV is a well-established indicator of cardiac autonomic activity mediated by changed parasympathetic and sympathetic levels [7]. These two competing branches of the autonomic nervous system (ANS) simultaneously send signals to the heart and cause a fluctuation in heart rate. HRV could reflect the affective and physiological aspects of pathogenesis and is emerging as a possible descriptor of pain assessment. For example, post-laparotomy pain in mice was assessed by heart rate and HRV, where heart rate was increased, whereas interbeat interval and standard deviation of interbeat interval were decreased. These changes returned by analgesics injection, suggesting that these parameters were associated with pain [8]. HRV was increased by persistent inflammatory pain states in rats, and Poincaré plot descriptor and pNN18 represented robust indicators of pain [9]. Cardiovascular autonomic parameters such as blood pressure, heart rate, and high-frequency power were increased in the rat model of chronic constriction injury of the sciatic nerve [10]. HRV analysis has also been widely studied in clinical practice. An analgesia nociception index (ANI) has been developed for pain measurement during surgical procedures, where parasympathetic activity was evaluated by monitoring the HRV balance between the nociceptive stimulus and the level of analgesia [11,12,13]. Prolonged pain in newborn infants was assessed by using HRV analysis, and postoperative pain was associated with a decrease in high-frequency HRV [14]. Autonomic dysregulation with increased resting heart rate and reduced HRV has been evidenced in patients with chronic pain disorders, such as low back pain, neck–shoulder pain, and fibromyalgia [15]. These studies suggest that HRV could be a useful tool for assessing pain disorders.

Therefore, this study aimed to investigate whether cancer pain leads directly to dysregulation of the ANS and can be assessed by HRV. We monitored changes in time- and frequency-domain HRV parameters and physiological responses with cancer progression in a peritoneal metastasis mouse model. Two weeks after cancer induction, the effect of analgesic administration on the HRV in the cancer model was analyzed.

## 2. Materials and Methods

### 2.1. Animal

All animal experimental procedures were approved by the Institutional Animal Care and Use Committee of Korea Institute of Science and Technology (Certificate number: KIST-2021-06-073). The experimental protocol was performed in accordance with the recommendations for the care and use of laboratory animals. Thirteen BALB/C male mice (DBL Co., Ltd., Chungbuk, Korea) of 10 weeks of age were used for this study, and mice were individually housed in conditions of a controlled 12 h/12 h light/dark cycle with free access to food and water. All animals had a one-week adaptation period prior to surgery, and individuals with abnormal weight changes were excluded from the study through periodic weight measurement. All animals were randomly divided into two groups: naïve (*n* = 5) and cancer (*n* = 8).

### 2.2. ECG Electrode Fabrication and Implantation

**Figure 1:**
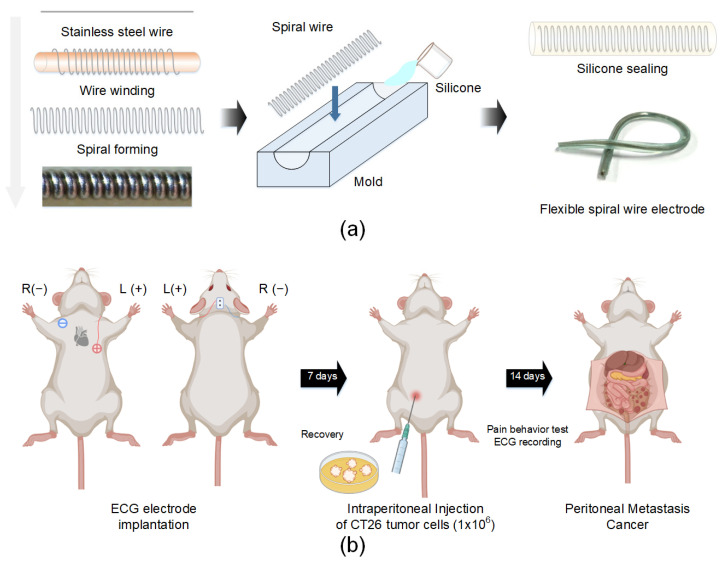
An overview of the experimental procedures and illustration. (**a**) Fabrication process of the flexible wire electrode. (**b**) Implantation of the ECG electrode in mice and the peritoneal metastasis cancer model.

A flexible wire electrode was fabricated to record electrocardiograms (ECGs) in a mouse model [16]. Figure 1a shows the fabrication process. A spiral shape was produced using a stainless-steel wire with a diameter of 0.1 mm. Then, the side end of the wire was fixed and uniformly wrapped around the stainless-steel fiber with a diameter of 0.86 mm. Medical-grade silicone (Ecoflex, 00-30) was mixed, and the air bubbles were removed in a vacuum chamber. The custom-made molds consisted of two side cap molds. After slowly injecting silicone into the molds, a spirally formed wire was placed in the center of the mold containing silicone. The molds were assembled, and then the silicone was cured at 80 °C for 60 min. The electrode was carefully separated from the molds and immersed in 70% ethanol under UV light overnight.

For electrode implantation, mice were anesthetized with 2% isoflurane and 0.9 L/min 100% O_2_. The hair of the head, neck, and chest was shaved. Two incisions were made in the left abdomen below the heart and the right upper shoulder, and two electrodes were fixed to the incised tissues. The opposite side of the electrodes subcutaneously led to the head connector. All incisions were sutured, and the head connector was secured to the skull using dental cement (Figure 1b).

### 2.3. Peritoneal Metastasis Cancer Model

Mouse colon carcinoma (CT-26) cells were purchased from the Korean Cell Line Bank (KCLB, Seoul, Korea). Fetal bovine serum (FBS), RPMI-1640 media, and antibiotic solution (1% penicillin and streptomycin) were purchased from Welgene Inc. (Gyeongsan-Si, Gyeonsangbuk-do, Korea). CT-26 tumor cells were maintained in RPMI-1640 medium supplemented with 10% FBS and 1% antibiotic solution at 37 °C in a humidified incubator containing 5% CO_2_. Seven days after electrode implantation, mice were administered an intraperitoneal injection of 1 × 10^6^ CT-26 cells.

### 2.4. ECG Recording and HRV Parameter Extraction

ECG was recorded for baseline measurements of 5 min every four days. ECG signals were digitized at a sampling rate of 32 kHz using a digital data acquisition system (Neuralynx, Tucson, AZ, USA). To reduce circadian and environmental effects on heart rate fluctuations, all recordings were performed at the same time and in an isolation recording chamber. All ECG signals were analyzed using MATLAB R2015b (MathWorks, Inc., Natick, MA, USA). Table 1 shows the formulas used to calculate HRV parameters [17,18]. First, ECG signals were bandpass filtered by a 3rd-order Butterworth of 0.5~50 Hz, and the R-peak was detected using the Pan–Tompkins algorithm with some modification [19]. The interval between two successive R-peaks (RRI) was accumulated over a 3 min time window for short-term ECG recordings. The mean R-R interval (mean RRI), square root of the mean squared differences of successive R-R intervals (RMSSD), and percentage of successive R-R interval differences greater than 5 ms (pNN5) were calculated for the time-domain analysis of HRV. For frequency-domain analysis, a tachogram was reconstructed at a 10 Hz sampling rate using cubic spline interpolation of the RRI, and the power spectrum was estimated by using Welch’s method. Low-frequency (LF) and high-frequency (HF) bands were set to 0.1–1.0 Hz and 1.0–4 Hz, and the ratio of LF and HF power (LF/HF) was analyzed.

### 2.5. Pain Assessment

To assess pain after CT-26 tumor cell injection, each of the four categories was evaluated in both the naïve and cancer groups. Four categories were calculated based on each parameter for physiological characteristics, posture, appearance, and activity [8,20]. Table 2 shows normal and abnormal behaviors for pain assessment. Mice were assigned a score of 0 or 1 for each category.

Physiological characteristics of pain were defined as body weight, body temperature, and diarrhea. When the body weight was increased or decreased by more than 10% compared to the previous measurement or when body temperature exceeded the normal temperature range of 35.8~37.4 °C, it was determined to be a signal of physical abnormality. In addition, diarrhea or bloody stools were considered symptoms of positive pain reactions. The rough hair coat, pinched face, and distended abdomen were identified as parameters of an appearance pain index. In the posture evaluation, a hunched posture was regarded as abnormality. In the activity category, the activity was evaluated as decreased when mice did not move or grow for more than 30 sec within 5 min of the observation time. Pain assessment was performed from video clips recorded simultaneously with the ECG measurement. A score of 0 was assigned to normal mice, while a score of 1 was allocated for the corresponding category when a positive reaction for the parameter was observed in mice. Therefore, the most painful case was given a score of 4.

### 2.6. Analgesic Evaluation

To evaluate the effect of analgesics in a cancer model, the changes in HRV according to analgesic administration were analyzed 14 days after cancer induction. Tramadol is a centrally acting weak μ opioid agonist that has been widely used to treat both acute and chronic pain [20]. The cancer group was randomly assigned into two groups: the cancer+saline (CS, n = 4) group and cancer+tramadol (CT, n = 4) group, which included saline and 5 mg/kg tramadol, and ECG was recorded one hour after saline and tramadol injection.

### 2.7. Statistical Analysis

All data are expressed as the mean ± standard error of the mean (SEM), and SPSS Statistics V25 was used for statistical analysis. The Mann–Whitney test was used for statistical analysis of the pain assessment, while the Kruskal–Wallis test was used for statistical analysis of the differences in HRV. The Bonferroni correction method was used as a post hoc test. The statistical significance (*p*-value) of each section is indicated by * *p* < 0.05.

## 3. Results

### 3.1. Pain Assessment

**Figure 2:**
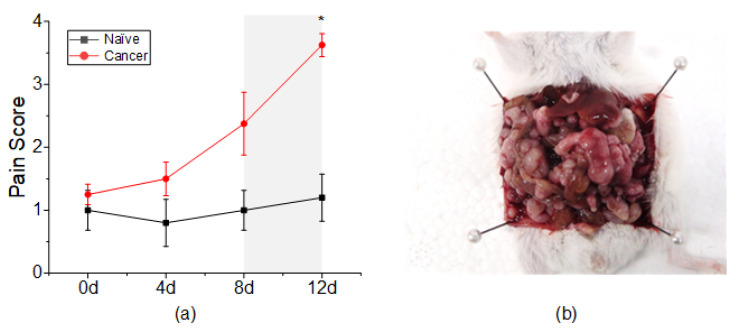
Pain assessment of naïve and cancer groups. (**a**) The pain score in the cancer group was significantly increased at day 12 compared to that in the naïve group. (**b**) Representative image of tumor formation at 12 days after CT-26 injection. * *p* < 0.05 vs. naïve.

The body weight of the cancer group was not noticeably different from the naïve group until day 8 but was significantly increased on day 12 (cancer vs. naïve, 30.9888 ± 0.9593 g and 27.98 ± 0.4492 g). Body temperature was also similar in the cancer group and the naïve group until day 8 but decreased significantly on day 12 (cancer vs. naïve, 35.6125 ± 0.3907 °C and 36.24 ± 0.02449 °C). Diarrhea symptoms could be observed on day 8 in most mice in the cancer group. The characteristics of appearance due to pain could be observed mainly in mice in the cancer group after day 8. In addition, low activity was observed in the cancer group on day 8. Tumor formation was visually confirmed by laparotomy of the cancer group mice. The tumor formed a bundle of masses, like small beads, along the mesenteric membrane and colon (Figure 2b). The results of comparing the pain score between the cancer group and the naïve group are shown in Figure 2a. The pain score of the cancer group was significantly increased at day 12 (cancer vs. naïve, 3.625 ± 0.1830 and 1.2 ± 0.3742). From these results, we confirmed that pain occurred 12 days after injection of CT-26 tumor cells in our experiments.

### 3.2. Changes in HRV According to Cancer Induction

**Figure 3:**
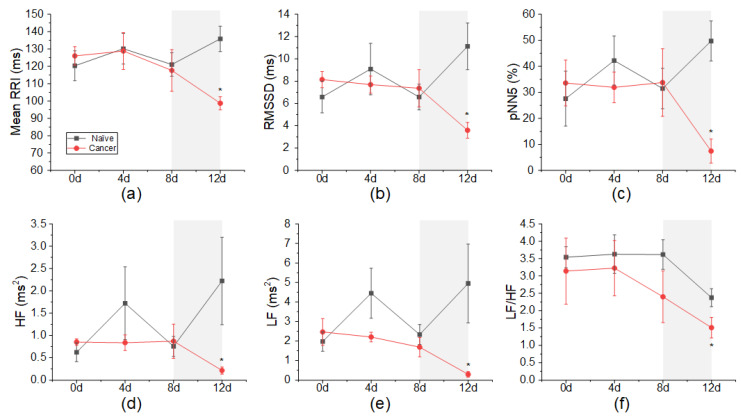
Effects of cancer pain on heart rate variability parameters. (**a**) MeanRRI. (**b**) RMSSD. (**c**) pNN5. (**d**) HF. (**e**) LF. (**f**) LF/HF. All HRV parameters in the cancer group were similar to those in the naïve group until day 8 but significantly decreased at day 12. * *p* < 0.05 vs. naïve.

Figure 3 shows HRV parameters calculated from the RRI during the 3 min ECG recordings. The time-domain HRV parameters, mean RRI, RMSSD, and pNN5, were similar in the cancer group and the naïve group until day 8 but significantly decreased at day 12 (mean RRI: cancer vs. naïve, 98.6941 ± 3.8106 ms and 135.8685 ± 7.3151 ms/RMSSD: cancer vs. naïve, 3.5971 ± 0.7143 ms and 11.1277 ± 2.0890 ms/pNN5: cancer vs. naïve, 7.4111 ± 4.6096 % and 49.7319 ± 8.5886 %). Generally, HF is regarded as an index of parasympathetic activity, and LF reflects both sympathetic and parasympathetic activity [21]. The total power of the cancer group decreased over time, and HF and LF were significantly lower at day 12 compared to the naïve group (HF: cancer vs. naïve, 0.2134 ± 0.07924 ms^2^ and 0.002221 ± 0.9787 ms^2^/LF: cancer vs. naïve, 0.2926± 0.1373 ms^2^ and 4.950 ± 2.020 ms^2^/LF/HF: cancer vs. naïve, 1.5099 ± 0.2970 and 2.3742 ± 0.2595).

### 3.3. Changes in HRV According to Analgesic Administration

**Figure 4:**
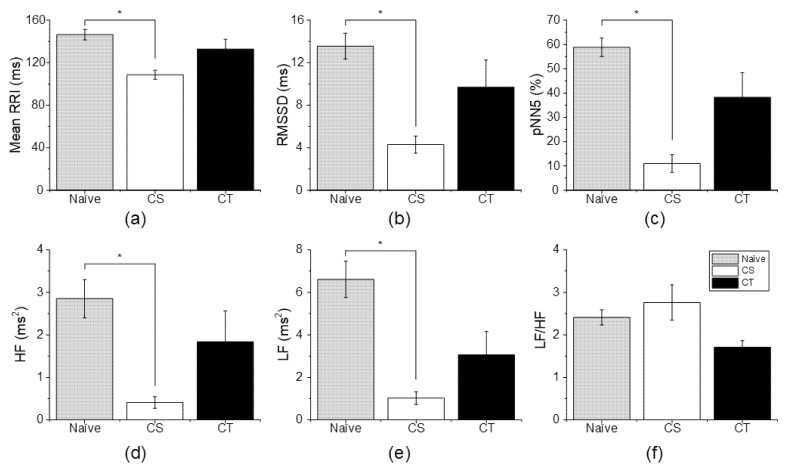
Effect of analgesic administration on heart rate variability parameters. (**a**) MeanRRI. (**b**) RMSSD. (**c**) pNN5. (**d**) HF. (**e**) LF. (**f**) LF/HF. All HRV parameters except LF/HF returned to normal after analgesic administration. * *p* < 0.05 vs. naïve.

To confirm the relationship between cancer-induced HRV changes and pain, an analgesic administration experiment was performed. As shown in Figure 4a, the mean RRI of the CS group was significantly decreased compared to that of the naïve group (CS vs. naïve, 108.6370 ± 4.2429 ms and 146.3713 ± 4.9446 ms), while the mean RRI of the CT group returned to a normal range, and there was no significant difference between the CT group and naïve group (CT vs. naïve, 132.8444 ± 9.1838 ms and 146.3713 ± 4.944 ms). For RMSSD and pNN5, the CS group exhibited a significant decrease compared to the naïve group (RMSSD: CS vs. naïve, 4.2941 ± 0.8068 ms and 13.5546 ± 1.2257 ms/pNN5: CS vs. naïve, 11.0152 ± 3.6618% and 58.8082 ± 3.8077%), but the CT group did not show a significant difference compared to the naïve group (RMSSD: CT vs. naïve, 9.6906 ± 2.5668 ms and 13.5546 ± 1.2257 ms/pNN5: CT vs. naïve, 38.2499 ± 10.1560% and 58.8082 ± 3.8077%) (Figure 4b,c).

In frequency-domain HRV parameters, HF and LF in the CS group were significantly decreased compared to the naïve group (HF: CS vs. naïve, 0.411 ± 0.138 ms^2^ and 2.851 ± 0.451 ms^2^/LF: CS vs. naïve, 1.025 ± 0.2957 ms^2^ and 6.610 ± 0.8546 ms^2^). The HF and LF in the CT group were decreased compared to those in the naïve group, but there was no significant difference (HF: CT vs. naïve, 1.837 ± 0.7267 ms^2^ and 2.851 ± 0.451 ms^2^/LF: 3.066 ± 1.095 ms^2^ and 6.610 ± 0.8546 ms^2^) (Figure 4d,e). For LF/HF, the CS group and CT group showed no difference compared to the naïve group (CS vs. naïve, 2.770 ± 0.4171 and 2.4116 ± 0.1767/CT vs. naïve, 1.710 ± 0.1548 and 2.4116 ± 0.1767).

## 4. Discussion

Pain acts as a serious psychological and physical stress and is one of the main factors that reduces quality of life [2,22]. Pain increases the body’s stress response and leads to ANS dysfunction. HRV is regulated by the ANS and could be a noninvasive indicator of ANS activity. Therefore, HRV analysis is widely used as a tool to assess ANS activity, and various studies have been conducted to monitor pain through evaluation of the ANS [7,23]. For example, postoperative pain in newborn infants is associated with a decrease in high-frequency HRV [14]. Children with chronic pain have significantly lower resting HRV and a sustained stress response with minimal variability in response to new acute pain stressors [24]. In stress-related chronic neck pain, HRV biofeedback increases resting HRV and enhances beneficial effects on ANS regulation, thereby reducing self-rated pain [15]. In this study, changes in HRV according to cancer progression were analyzed in a peritoneal metastasis mouse model for pain assessment. The pain scores of mice in cancer, including physiological characteristics, posture, activity, and appearance, were evaluated as physiological responses to pain, and they significantly increased on day 12 compared to the naïve group. In addition, time- and frequency-domain HRV parameters were analyzed at the same time and showed a significant decrease on day 12. Low HRV could reflect reduced parasympathetic activity, which is associated with poor health outcomes such as cardiovascular disease, mood disorders, behavioral disorders, and mortality [21,25]. These results demonstrated that cancer pain resulted in a significant decrease in parasympathetic activity.

To investigate whether low HRV is an effect of cancer-induced pain, we observed changes in HRV caused by analgesics. Tramadol enhances the inhibition of pain transmission through the modulation of neurotransmitters that act as μ-opioid agonists, and these properties can induce analgesic effects [20,26]. When the changes in HRV were compared with and without tramadol administration, the low HRV induced by cancer progression was increased to a similar level in the naïve group. Overall ANS activity was decreased by cancer pain, while all HRV parameters except LF/HF returned to normal after tramadol administration. These observations suggest that HRV analysis can be used to assess cancer pain associated with peritoneal metastasis.

A limitation of this study is that it is difficult to generalize cancer pain assessment because the number of experimental animals is small. In addition, the symptoms of pain in the cancer animal model used in the study were unclear compared to other pain models, such as neuropathic pain. We assessed the pain score by observing the changes based on the four categories considering changes in body and behavior caused by cancer. These signs of pain are expressed as long-term consequences of cancer progression (e.g., body weight change, diarrhea, distended abdomen/swollen, etc.). We used tramadol as an analgesic, and the effects of analgesic administration are temporary. Therefore, it was difficult to confirm the symptoms of pain reduction by analgesics in the case of cancer pain. In addition, ECG was transmitted to the data acquisition system by wire through the head connector. HRV is affected by environmental changes and is sensitive to stress. Therefore, the number of ECG recordings should be minimized because such a measurement environment can give stress to mice. Unfortunately, pain score and HRV analysis were not assessed after analgesic injection. In the future, we will consider ECG recording in the free-moving state using an implantable transmitter and will study changes in HRV and pain score by analgesics in various pain-related diseases.

Pain is difficult to generalize because it is a very subjective experience that is emotional and sensitive [5,27]. Individual differences in HRV are also common. In addition, the relationship between the parasympathetic and sympathetic branches is complex, and HRV indexes interact with ANS processes in both linear and non-linear ways. Many studies have proposed an adjusted approach for interpreting and using HRV to achieve a better indicator of vagal activity and to account for the interrelationships between HRV and heart rate [28,29]. Furthermore, non-linear measurement indexes such as S, SD1, SD2, approximate entropy, sample entropy, detrended fluctuation analysis, and principal dynamic mode have been suggested to represent the complexity of HRV and have yielded useful results [8,9,30,31]. Although our experiment confirmed that the time- and frequency-domain HRV parameters were decreased by cancer pain, we believe that a more accurate interpretation may be possible through analysis of modified HRV or non-linear HRV parameters. Further studies will perform more experiments and analyses.

## Figures and Tables

**Table 1:** Descriptions and formulas used to calculate heart rate variability parameters.

Parameter	Unit	Formula	Description
Time-domain HRV parameters			
MeanRRI	ms	$t\frac{\sum_{i=1}^{N}RR_{i}}{N}$	Mean variability of interbeat interval
RMSSD	ms	$\sqrt{\frac{\sum_{i=1}^{N-1}{\left(RR_{i+1}-RR_{i}\right)}^{2}}{N-1}}$	Reflects parasympathetic activity
pNN5	%	$\frac{Count(\left(RR_{i+1}-RR_{i}\right)>5\;\mathrm{ms})}{N-1}\times 100$	Reflects parasympathetic activity
Frequency-domain HRV parameters			
LF	ms^2^		Reflects both parasympathetic and sympathetic activity
HF	ms^2^		Reflects parasympathetic activity
LF/HF			Reflects parasympathetic and sympathetic balance

**Table 2:** Categories used to calculate pain.

Categories	Pain Sign Parameter (+/−)
Physiological characteristics	10% or more body weight change Above normal body temperature (35.8~37.4 °C) Diarrhea
Posture	Hunched posture
Appearance	Rough hair coat Pinched face Distended abdomen/swollen Reluctance to move
Activity	Not grooming

## Data Availability

The data used in this study are available on request from the corresponding author. The data are not publicly available because of participant confidentiality.
